# The Biological Basis and Analyses of N-Glycan Microheterogeneity

**DOI:** 10.1016/j.mcpro.2025.101491

**Published:** 2025-12-17

**Authors:** Trevor M. Adams, Peng Zhao, Sree Hari Seenivasan, Lance Wells

**Affiliations:** Department of Biochemistry and Molecular Biology, Complex Carbohydrate Research Center, University of Georgia, Athens, Georgia, USA

**Keywords:** N-glycosylation, microheterogeneity, mass spectrometry, glycoproteomics, N-glycan synthesis

## Abstract

N-glycosylation is an abundant and essential co/post-translational modification that is preserved across all eukaryotes. N-glycans have important functions in protein stability and protein–protein interactions. N-glycans exhibit a high degree of heterogeneity, even within an individual site on the same protein, a phenomenon that is termed “microheterogeneity,” which is the focus of this review. Traditional analytical approaches with released glycans are limited in their usefulness in studying microheterogeneity because of most glycoproteins having more than one site of N-glycosylation. Since specific N-glycans at specific sites can confer important functions to glycoproteins, this presents a significant gap between the information content of glycomics and glycoproteomics experiments. More recently, tandem mass spectrometry of intact glycopeptides has been used to obtain site-specific information on N-glycan microheterogeneity. The microheterogeneity of glycoproteins presents a significant analytical challenge not only during mass spectrometry analyses but also in downstream data processing. Use of specialized search engines followed by extensive manual validation is often required for accurate and in-depth glycoproteomics. Overall, recent advances in analytical technology and data processing present exciting new opportunities to analyze N-glycans in a site-specific manner. Being able to define, understand functional consequences of, and even predict and direct N-glycan microheterogeneity has implications across many fields, including the manipulation and production of glycoprotein biologics.

## Highlights

• N-glycans are critical for glycoprotein structure and function.
• N-glycan microheterogeneity is a site-specific property of glycoproteins.
• Historical overview of N-glycan processing and enzyme specificities.
• Overview of current methods and challenges in glycoproteomics.

## Graphical Abstract

**Figure:**
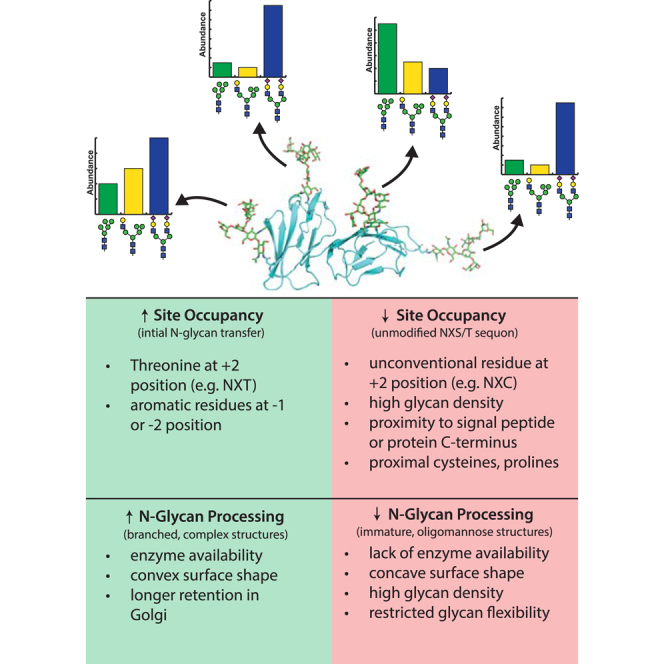

## In Brief

Unlike most other post-translational modifications, N-linked glycosylation encompasses a plethora of different structures and is almost always heterogenous at each site of modification. The presence of multiple glycans at the same site of glycosylation on a specific protein is referred to as “microheterogeneity” and is believed to be influenced by the local structure near the site of the modification and likely arises from incomplete processing coupled with divergent glycosylation pathways. N-glycan microheterogeneity has implications in protein structure, function, and the production of biologics/biosimilars. The current primary method for analysis consists of mass spectrometry followed by data processing by specialized search engines.

N-linked glycosylation is an essential eukaryotic co/post-translational modification that is required for proper embryogenesis and glycoprotein stability (1). A significant amount of cellular resources are dedicated to the production of N-glycans and their nucleotide sugar precursors, including >1% protein-encoding genes for glycosyltransferases in higher eukaryotes (2). One of the reasons why glycan processing is crucial to higher eukaryotes is that it serves to distinguish host tissue from nonhost cells, which often present their own distinct classes of glycans (3). For this reason, it is both a clinical and regulatory priority that glycosylation of manufactured biologics has consistent, human-compatible glycosylation, whether these biologics are produced in mammalian, insect, or even yeast cell lines (4, 5, 6). Furthermore, shifting the type of glycans presented on a glycoprotein can improve desired properties of certain biologics (7). Thus, it is important that we be able to define and eventually be able to predict and manipulate N-glycan microheterogeneity by understanding the biological basis of the process. It should be noted that O-glycans also have microheterogeneity that modulates function, but this topic is beyond the scope of this review.

**Fig. 1:**
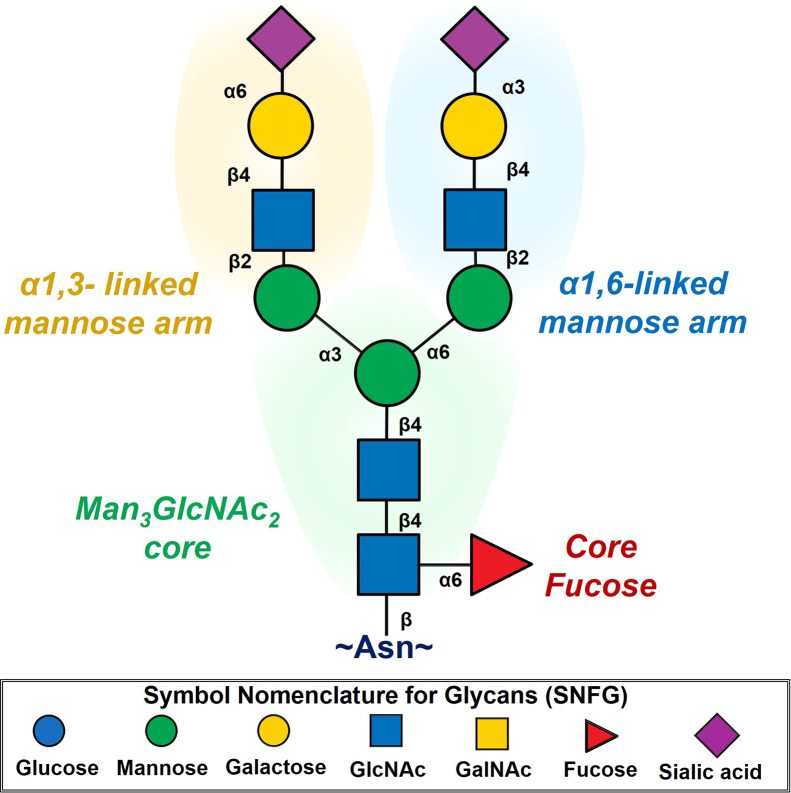
**Anatomy of a biantennary complex N-glycan**. The ⍺1,3-linked arm and ⍺1,6-linked arm extend out from the chitobiose core, which may or may not be core fucosylated. Glycans are represented using the Symbol Nomenclature for Glycans (SNFG). The conserved core consists of two GlcNAcs extended by a trimannose fork. All the mannoses can be further extended by a GlcNAc residue, and the ⍺1,3-linked arm and ⍺1,6-linked mannoses can be further branched by GlcNAc residues (not shown) as well as further elaborated (shown here as a simple but common example of Gal-Neu5Ac extension in two different linkages).

Due to their size, diversity, and numerous biological functions, N-glycans have been a point of focus for glycobiologists for several decades. The array of structures present, as well as the rules for assembly, can vary greatly between species. In this review, we will focus almost exclusively on N-linked glycosylation in humans. N-glycans are initially transferred *en bloc* to asparagines within the conserved sequence Asn-X-Ser/Thr (termed “sequons”) (8), where X is any amino acid except proline. The presence of a sequon is usually necessary but not sufficient for N-glycan transfer (9); however, there are exceptions, such as addition of N-glycans to Asn-X-Cys groups, at a lower frequency (10). In addition, there are occasional observations of N-glycosylated nonconsensus motifs, including Asn-X-Val (9, 11), Asn-X-Gly (12), Asn-X-Asn (13), and Asn-X-Gln groups (14). This suggests that the oligosaccharyltransferase (OST) is able to accommodate a variety of amino acids at the +2 site but has clear preferences for Ser/Thr (15). However, it is also important to note that explicit confirmation of novel N-glycosylation sites *via* intact glycopeptide analysis is required, as artifacts generated during sample preparation or data analyses can produce misleading results (16). The sugars that predominate in human N-glycans are mannose (Man), GlcNAc, galactose (Gal), GalNAc, *N*-acetylneuraminic acid, glucose, and fucose (Fuc) (1, 17). Other monosaccharides, such as *N*-glycolylneuraminic acid (Neu5Gc), incorporated into a sugar nucleotide solely by the salvage pathway from diet, glucuronic acid, and xylose, are rarely observed on human N-glycans, though can be common in other species and other types of glycans (18) (Fig. 1). The donor species for sugar transfer are nucleotide sugars, such as UDP-GlcNAc, GDP-Fuc, or CMP-*N*-acetylneuraminic acid (19). N-glycans have a common Man_3_GlcNAc_2_ core structure that forms a minimum of two branches. There are three major classes of N-linked glycans that refer to extensions and branching beyond the core structure. Oligomannose N-linked glycans are generated in the endoplasmic reticulum (ER) with both branches containing Man residues. These can be modified so that the three-linked arm is modified by GlcNAc and potentially further elaborated, whereas the six-linked arm retains Man residues that are referred to as hybrid N-linked glycans. Finally, both arms can be modified by GlcNAc that can be further branched from the 3,4- and/or 6-linked Man, and the GlcNAc moieties further extended with additional monosaccharides that we refer to as complex (1). The reducing-end GlcNAc, which is attached to the Asn side chain, can also be fucosylated, a phenomenon termed “core fucosylation” (Fig. 1). ⍺1,6-linked core fucosylation of the Asn-bonded GlcNAc by the fucosyltransferase FUT8 often occurs only on hybrid and complex N-glycans (20), although interestingly, there are exceptions with certain fully folded proteins (13) resulting in fucosylation of oligomannose N-glycans (14, 21).

While a full understanding of N-glycan synthesis, branching, and maturation is critical to understanding the diversity of potential glycoforms, this topic is beyond the scope of the review. We instead direct interested readers to a review by Schachter (22), which goes over forks in glycan processing with great detail, and a review by Moremen *et al*. (23) that focuses on diversity and synthesis of vertebrate protein glycosylation, in addition to Essentials of Glycobiology, Fourth Edition, Chapter 9 (1).

## Historical Perspective

The earliest references to microheterogeneity in the literature registered in PubMed refer to protein microheterogeneity because of small distributions of differential activity, size, or separation within a purified sample of protein (24). In some of these cases, it was discovered that the underlying source of these differences was due to sugar modifications creating subpopulations of glycoproteins (25, 26). The term “microheterogeneity” began being applied to glycans as advances in glycan characterization developed during the 1960s, eventually becoming a term that referred to the high number of possible structural variants that can exist at any given site on a glycoprotein (18). The term microheterogeneity (the complexity of structures that exist at a given site of glycosylation) should not be confused with the term macroheterogeneity, which refers to the variable occupancy of glycans at a potential site of modification. However, it is crucial to recognize that a site that is not always occupied increases the number of potential states of any given sequon by one for the unglycosylated state. Thus, variable site occupancy, macroheterogeneity, must be integrated into comprehensive glycosylation analyses, as it is a critical and sometimes overlooked component of the overall structural complexity of a glycoprotein population.

Early studies in the 1960s of readily obtained glycoproteins, such as ovalbumin (27, 28) and fetuin (29) demonstrated common linkages between many of the observed sugars as well as variations in the total composition of the glycans. Later experiments with thyroglobulin by Spiro (30)investigated microheterogeneity and found that the glycoprotein contained two distinct glycan units, which were separable with extensive dialysis. Furthermore, it was demonstrated that much of this heterogeneity could be homogenized with neuraminidase treatment, demonstrating the contribution of sialic acids (Sias) to observed protein heterogeneity in electrophoresis (30, 31). Along with studies characterizing ovalbumin (32, 33), immunoglobulin G (IgG) (34), and other proteins (26, 35, 36, 37, 38, 39, 40, 41, 42) it was eventually recognized that this heterogeneity was likely because of incomplete processing by glycosyltransferases (43, 44, 45), and that this incomplete processing is likely influenced by protein configuration (39).

In the early 1970s, new analytical techniques such as isoelectric focusing were applied, and additional proteins were shown to demonstrate neuraminidase-sensitive microheterogeneity (46, 47, 48). It was also shown that the carbohydrate content of glycoproteins in mouse embryos may be altered over the course of development (49) because of an increase in sialyltransferase activity (50). The various specific endoglycosidases and exoglycosidases also became an invaluable tool for carbohydrate identification during this time (51). Work by the Kobata group eventually led to the elucidation of oligomannose structures (52), characterization of hybrid structures (53), and elucidation of complex biantennary N-glycan structures (54), a notable step forward from the compositional analysis of the 1960s. At this time, enough site-specific N-glycan data became available that it became possible to look at general trends of N-glycans within the context of the glycoprotein’s primary structure. Pollack (55) and Atkinson found that oligomannose glycans tended to be more common toward the C-terminal end of proteins, suggesting that protein folding may impact the accessibility of N-glycans, a finding that was supported by other statistical studies (56).

Mass spectrometry (MS) combined with methylation (57) and chromatographic separation also took several steps forward at this time that facilitated analyses of glycans and glycoproteins. Fast atom bombardment–mass spectrometry (FAB–MS) and glycosidase treatment were used to determine the structure of N-linked lactosaminoglycans (58, 59). FAB–MS was used in conjunction with neutral gas collision in order to generate fragments that were informative of oligosaccharide structure, even between isomeric groups (60). FAB–MS, combined with endoglycosidase H (Endo H) treatment and classical peptide separation techniques, was shown to be capable of deducing which sequons were occupied on a multiply glycosylated yeast invertase (61). Electron impact-field desorption MS was used to determine the structure of larger oligomannose N-glycans such as Man_8_GlcNAc_2_ (62). NMR analysis was also used to identify less populous species that contribute to N-glycan microheterogeneity in ovalbumin (63). While all these approaches have contributed greatly to our understanding of N-glycans and glycoproteins in general, it is important to note that many of them do not consider site-specific N-glycosylation, in which detailed analysis has only been recently made possible with the development of new analytical techniques (see section “*Site-Specific Analysis of N-Glycans*”).

## Biological Determinants of N-Glycan Microheterogeneity

### Macroheterogeneity: Efficiency of Core Glycosylation

The initial transfer of the Glc_3_Man_9_GlcNAc_2_ to the Asn-X-Ser/Thr sequon on acceptor glycoproteins by OST is a key step in producing heterogeneity in that incomplete transfer, which is a common occurrence, will result in a subpopulation of unglycosylated sequons. Incomplete site occupancy of N-glycan sequons has been termed “macroheterogeneity” (64). The proportion of occupied *versus* unoccupied sites can vary drastically even on a single glycoprotein, as illustrated with the ranging occupancies of HIV envelope (Env) glycoprotein (65). For some glycoproteins, the specific nature of the glycans themselves is often of less importance than their general presence, so their macroheterogeneity may have a greater impact on function than microheterogeneity. For viral glycoproteins like influenza A virus hemagglutinin, glycans shield lysines, arginines, and aromatic residues that are the target of defensive host proteases like trypsin and chymotrypsin for delivery of glycopeptides to the major histocompatibility complex (66, 67). Indeed, inhibition of glycosylation with tunicamycin increases the rate of influenza nucleoprotein degradation (68). However, for complex organisms, processing of the glycans can be important for proper development. MGAT1 knockout in mice is embryonic lethal, indicating that the formation of hybrid and complex N-glycans rather than the presence of N-glycans in general is essential for higher eukaryotic development (69). Complex-type N-glycans are expressed as early as the four-to-eight cell stage of embryonic development, and mouse embryos cultured with the OST inhibitor tunicamycin fail to develop past 11 days (70).

As this initial step of N-glycosylation usually happens cotranslationally in higher eukaryotes (exceptions discussed later), it occurs on the unfolded nascent polypeptide rather than a mature, folded glycoprotein. Transfer of the oligosaccharide by OST takes place within 30 to 40 Å of the ER lumen, which is roughly the length of 12 to 14 amino acid residues (71). For this reason, it is likely that in some cases, core glycosylation may be in competition with protein folding in the context of secondary structure formation. This is supported by the poor glycosylation efficiency found at sequons that are in close proximity to cysteine residues known to participate in disulfide bonds, and limiting disulfide bond formation has been demonstrated to promote glycosylation at these sites (72).

The primary sequence of glycoproteins seemingly has the most impact on glycans at the point of core glycosylation. The presence of a threonine rather than a serine in the +2 position of the sequon increases the efficiency of core glycosylation of rabies virus glycoprotein, although it is unclear if or how this impacts further processing by Golgi enzymes (73). Also, roughly 75% of unoccupied sequons have a serine in the +2 position, further supporting the idea that threonine is preferred at the +2 position for efficient transfer (74). Sequons with aromatic residues in the −2 and −1 positions are more likely to be heavily glycosylated (74). The presence of proline in either the “X” position or immediately C-terminal to the hydroxy-amino acid at +2 abrogates core glycosylation (75). In further studies with rabies virus glycoprotein, it was shown that the “X” moiety can have some impact on site occupancy as large, hydrophobic residues such as tryptophan appear to reduce the efficiency of core glycosylation (76). Close proximity to the signal sequence may also be responsible for poor core glycosylation efficiency (77). On the other end of the nascent peptide, C-terminal N-glycans are thought to interact more weakly with STT3A (OST-A) because of the formation of secondary and tertiary structures as the protein nears full synthesis, and these C-terminal sequons are thought to be modified post-translationally primarily by STT3B (OST-B) and are often more substoichiometric (15). All these negative effects on core glycosylation tend to be more pronounced at sequons that contain a serine rather than a threonine in the +2 position (74).

The macroheterogeneity of a glycoprotein is often resistant to changes in the expression system. Two recombinant proteins studied with varying expression conditions in Chinese hamster ovary cells demonstrated only marginal changes in site occupancy as temperature, pressure, pH, and metal ion concentration were varied (78). Some viral glycoproteins have also been shown to have comparable site occupancies when expressed across different cell lines (79, 80). Therefore, it seems that while glycan diversity can be dramatically impacted by expression conditions, site occupancy is comparatively robust, likely because of being largely defined by the activity of a single enzyme (OST). Moving forward, knowledge of primary and tertiary structural features that help define macroheterogeneity could be utilized to more effectively engineer novel sites of N-glycosylation into glycoprotein targets, including biologics. This type of analysis should benefit greatly from large-scale site mapping studies such as those conducted by Mann *et al*. (9)in mice as well as unbiased large-scale quantitative occupancy studies in organisms.

### Organism-Specific Considerations

While N-glycosylation is broadly conserved across metazoans, there is significant evidence that the organism in which a protein is expressed can have a dramatic effect on its glycosylation. Studies of γ-glutamyl transpeptidase by the Kobata group revealed interspecies differences in transpeptidase glycosylation between rats, cattle, and mice (81). An interesting finding was the presence of bisected N-glycans in mouse and human kidney that were absent in bovine and rat kidney γ-glutamyl transpeptidase (82, 83). A study of viral glycoproteins demonstrated significant differences in carbohydrate composition depending on the host cell that the viral glycoprotein was expressed, and site-specific analysis of trypsin-digested peptides revealed that this composition was due to diversity within individual sites (84). A comparison of bovine and human fetuin (hFetuin) showed similarities at shared sites but slight differences in branching preferences (85). The magnitude of these organism-specific differences may be tied to the phylogenetic proximity of the species, with more distal organisms differing more than closely related organisms, both because of the presence of additional sugars as well as altered enzyme specificities and expression levels. For example, when bovine trypsin (a normally unglycosylated protein) was expressed in maize, the protein presented N-glycans at several nonconsensus N-glycosylation sites (13).

Interspecies differences in N-glycosylation are important in the production of biologics, as nonhuman expression platforms (which have their own advantages) can produce immunogenic glycans. The insect glycome is enriched in paucimannosidic glycans (86). In contrast, the yeast glycome is often hypermannosylated, far exceeding what is seen in vertebrates (87). *Pichia pastoris* presents an exception with lower levels of hypermannosylation, which makes the yeast an intriguing platform for protein expression. Genetic engineering approaches can be used to reduce the differences in glycosylation between species, including eliminating Gal-⍺-Gal and Neu5Gc that are immunogenic in humans, and there is much interest in the production of glycoengineered “humanized” cell lines (88, 89).

**Fig. 2:**
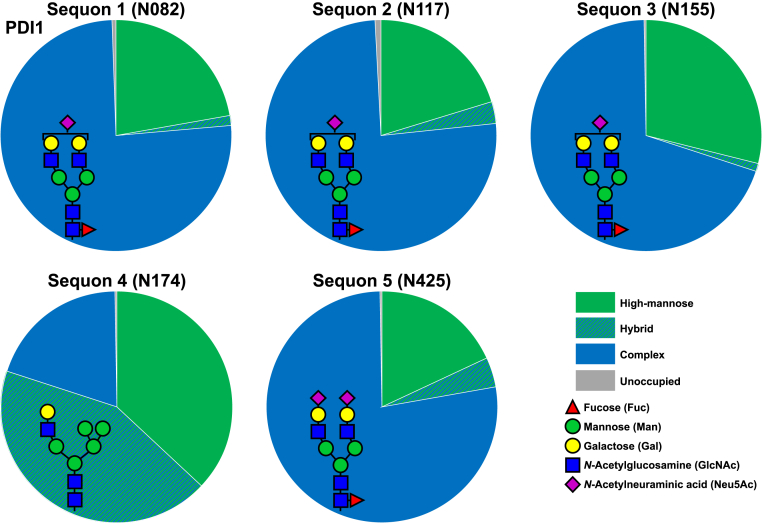
**Profiles of N-glycan types at five sequons of protein disulfide isomerase 1 (PDI1)**. The different profiles of N-glycan types show different levels of N-glycan processing at different sequons of PDI1. The most abundant glycan topology for the dominant glycan type at each sequon is displayed as the *cartoon insert*.

One way to deduce the role of species on N-glycan microheterogeneity is to perform glycopeptide analysis on a recombinant protein expressed in cell lines from different organisms. The glycoprotein protein disulfide isomerase 1 (PDI1) (Fig. 2, (90)) is easily expressed in high titers, is amenable to bottom–up MS approaches, and has five sites of glycosylation with only one that usually displays oligomannose structures (90, 91, 92). Oligomannose structures are displayed at this site, and N-glycan processing is quite slow regardless of whether the protein is expressed in human (human embryonic kidney 293F) or insect *Trichoplusia ni* cells. So, while species can have a great impact on complex N-glycan elaborations, the overall extent of N-glycan processing to complex glycans is often preserved, likely because of the overall accessibility of the glycan to highly conserved processing enzymes that convert N-glycans from oligomannose to hybrid to complex glycans.

### Cell Type

Cell type has been shown to impact N-glycan microheterogeneity. Early studies with vesicular stomatitis virus showed differences in glycan composition when the vesicular stomatitis virus glycoprotein was expressed in different cell types (93). Thy-1 (94) and OX2 (95) glycoproteins purified from rats have different carbohydrate compositions when purified from brain *versus* thymocytes. Purified pig endopeptidase-24.11 has been shown to have differential glycosylation when purified from the intestine rather than the kidney, particularly with respect to Fuc content (96). However, many of these early studies lacked the instrumentation necessary to deduce site-specific glycan data for these multiply N-glycosylated proteins and were thus limited to glycoprotein-specific information rather than site-specific information and the majority of the observed differences are likely explained by the extent and types of extensions from complex branched N-glycans.

Studies of γ-glutamyl transpeptidase by the Kobata group revealed interorgan differences in the glycosylation of the protein expressed in liver *versus* kidney, with the liver glycans all containing Sia groups and the kidney all neutral (97). Additional studies with Thy-1, this time empowered by NMR spectroscopy, demonstrated that thymus-expressed Thy-1 is sialylated, whereas brain-expressed Thy-1 is not (98). Interestingly, neural-expressed Thy-1 appears to have strong conservation of glycan-type across species, even though there are significant interspecies differences at the polypeptide level (99). Glycans isolated from brain tissue generally seem to differ substantially from other tissues, as mouse liver N-glycans are rich in Neu5Gc groups, whereas these are sparse in mouse brain (100). Using Sindbis virus glycoproteins, Hakimi and Atkinson (101)showed that related glycoproteins translated in the same compartment can result in differential glycosylation of oligomannose glycopeptides, suggesting the required mannosidases have varying activities toward glycan substrates.

A comparative analysis of native human and recombinantly expressed fetuin clearly demonstrates the impact that cell type can have on post-translational modifications. Lin *et al*. (85)expressed recombinant hFetuin in human embryonic kidney 293 cells and used a combined native and bottom–up MS approach to compare it to hFetuin from serum, which is mostly synthesized in the liver. They found that recombinant hFetuin was more likely to express terminally galactosylated and core fucosylated structures, whereas hFetuin expressed mostly sialylated structures. Comparison of closely related major histocompatibility complexes using radiolabeled sugars and site-specific release of glycans demonstrated that small structural variations can have dramatic consequences on the extent of branching found in complex glycans, and that glycosylation patterns are reproducible when expressed in the same tissue over time (in the case of this study, several months) (102).

### Protein Secondary Structure

N-glycosylation is most frequently found on β-turns (9, 103) where the underlying peptide backbone remains readily accessible after folding is complete (104). In addition, sites of N-glycosylation are enriched at points in which the secondary structure changes (74). Given that the initial transfer of the N-glycan precursor occurs early, often cotranslationally, the presence of the large, hydrophilic glycan moiety on the nascent chain may actively influence subsequent protein folding by stabilizing certain local conformations. This influence raises the question as to whether glycosylation promotes the formation of these secondary structures, such as β-turns, or restricts local folding dynamics in a way that correlates with the observed structural breakpoints. Interestingly, N-glycans are also enriched on β-sheets when compared with their distribution across proteins generally (105).

Time-resolved fluorescence energy transfer has been used on glycopeptides to demonstrate that glycosylation can affect the conformations that the peptide is able to adopt, indicating that N-glycosylation alters folding and potentially acts as a nucleation event for secondary/tertiary structure formation (106). The requirement of N-glycosylation for proper glycoprotein folding is domain dependent, as has been shown with the HIV Env glycoprotein gp120, whose V1 and V2, but not V3, domains require glycosylation for proper folding, likely because of recognition by calnexin–calreticulin chaperones (107).

### Protein Tertiary Structure

Initial N-glycan transfer to the sequon must occur cotranslationally or early post-translationally when the site is fully accessible to the OST complexes. Once the glycan is attached, the rapid folding of the polypeptide chain can create a local structural environment that sterically shields the new N-glycan. This folding can lead to the formation of protected packets that prevent subsequent processing by ER and Golgi mannosidases and glycosyltransferases, thereby locking the glycan in an oligomannose state and even shielding it from Endo H endoglycosidases (108). A study by Lee *et al*. (109)found a positive correlation between N-glycan processing and asparagine accessibility that provides more support for the local environment being a determining factor in glycan maturation.

A common example of protein structure impacting N-glycan processing is found in the glycosylation of IgG heavy and light chains, where the heavy chain N-glycan is mostly unbisected biantennary complex, whereas the light chain N-glycan is predominantly bisected (110). The reason behind this is revealed by the crystal structure of serum IgG. The 3D structure shows that the Man⍺1–6Man linkage of the heavy-chain N-glycan is in a gauche–gauche conformation (⍵ = −60°) (111) which is not a valid conformation for the bisected MGAT3 product that solely adopts the *trans*-gauche conformation (⍵ = +180°) (112). Essentially, the light-chain N-glycan is free to adopt an ⍵ of −60° or +180°, whereas the heavy-chain N-glycan is restricted by the tertiary structure of the glycoprotein to −60°, restricting its processing. The interactions between IgG glycans and the underlying protein are of great interest as these protein–glycan interactions are important in receptor binding and inducing antibody-dependent cellular cytotoxicity. Modification of the region surrounding the heavy-chain N-glycan can improve the antibody’s overall stability (113). Modification of the protein–glycan interface of IgG can lead to more extensive processing of the N-glycan, inducing hypergalactosylation and hypersialylation (114). Replacement of certain hydrophobic residues on IgG Fc with alanine causes an increase in heavy-chain galactosylation and sialylation, demonstrating the role of the underlying protein in defining N-glycan heterogeneity (115). For more information on immunoglobulin site occupancy and glycan heterogeneity, please see the recent review by Čaval *et al*. (64).

Much work has been completed recently by the Aebi group in deducing the underlying basis of N-glycan microheterogeneity using the model protein yeast Pdi1p. Pdi1p has five sites of N-glycosylation, one of which (site 4) is much less processed than the others (Fig. 2), and molecular dynamics simulations indicate that the glycan at this site interacts with the protein backbone of Pdi1b (92). Upon removing the region of the protein that the glycan was found to interact with, ER processing of the glycan improved. The Aebi group has also developed an *in vivo* kinetics model of N-glycan processing using parallel reaction monitoring MS (116).

Recent work by our group has demonstrated that tertiary structure plays a critical role in dictating N-glycan microheterogeneity (90). We expressed a series of reporter glycoproteins in MGAT1 cells, resulting in an enrichment of the Man_5_GlcNAc_2_ glycoform at a total of 38 sites of glycosylation on proteins ranging from having 3 to 22 sites. We then used these purified glycoproteins as substrates for purified glycosyltransferases and MAN2A1 and quantified the relative ratios of substrates and products *via* tandem MS to determine the rate of the reactions over time. Sites that were enriched with less processed structures (*e*.*g*., oligomannose and hybrid) were found to be worse substrates for the enzymes than their more-processed counterparts on the same glycoprotein. These transfer rate differences were abrogated when the reporter glycoprotein was reduced and digested into glycopeptides prior to enzyme addition, indicating that the tertiary structure of the glycoprotein is a key player in microheterogeneity (90). Notably, this also falls in line with the Aebi group’s earlier suggestion that the shape of the protein surface may dictate rates of N-glycan processing, with particular regard to the surface convexity–concavity at the site of N-glycosylation (117).

The quaternary structure of glycoproteins has also been shown to impact N-glycan microheterogeneity, which is reasonable considering the additional restrictions on three-dimensional space that occur as multimers form. The proteins Mac-1 and LFA-1 both have identical β-chains and differing ⍺-chains at the polypeptide level, and both form multimers before reaching the Golgi. The N-glycosylation of the β-chain differs from each multimeric species, indicating that quaternary structure can impact N-glycan processing (118). In addition, quaternary structure likely has an impact on glycan editing in some biological processes outside the ER and Golgi, such as the removal of Sias from certain glycoproteins at the cellular membrane (119, 120).

### Precursor Availability

**Fig. 3:**
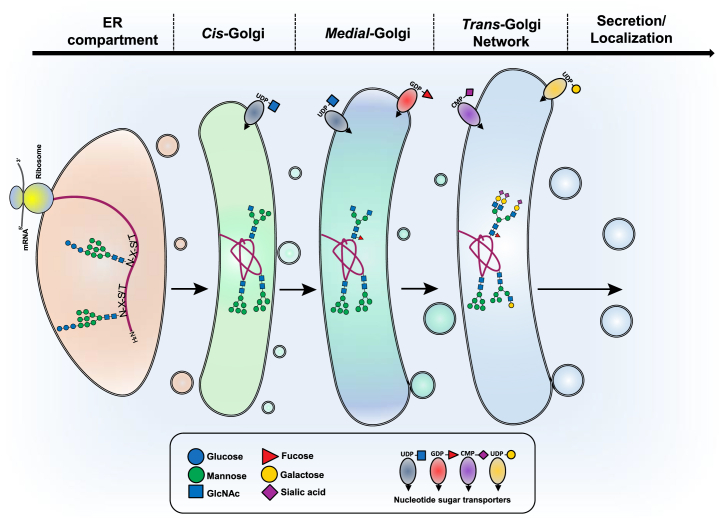
**N-glycan processing and maturation**. Following the transfer of Glc_3_Man_9_GlcNAc_2_ to protein, glucosidases in the endoplasmic reticulum (ER) remove three glucose residues, ER mannosidase removes a Man residue, and additional Man residues are removed in the *cis*-Golgi compartment until Man_5_GlcNAc_2_ is generated. The action of MGAT1 on Man_5_GlcNAc_2_ initiates the first branch of an N-glycan. Alpha-mannosidase II removes two outer Man residues and generates the substrate for MGAT2. The resulting biantennary N-glycan is extended by the addition of Fuc, Gal, and Sia to generate a complex biantennary N-glycan or can accept a GlcNAc from the branching enzymes MGAT4A, MGAT4B, MGAT4C, MGAT5, or MGAT5B before being elongated with Gal, GlcNAc, Sia, and Fuc. The sugar donors UDP-GlcNAc, UDP-Gal, GDP-Fuc, and CMP-Sia are marked by their location in the Golgi compartments. Fuc, fucose; Gal, galactose; Sia, sialic acid.

One potential source for N-glycan heterogeneity is the availability of nucleotide sugar donors. These precursors have relatively low concentrations in the cytosol (or nucleus in the case of CMP-Sia), where they are metabolized but are concentrated in the Golgi by nucleotide sugar transporters (121) (Fig. 3). Once inside the Golgi, the nucleotide sugars can be used as sugar donors for glycosylation. Degradation of these sugar donors is counterintuitively important for the proper cycling of metabolites because of nucleotide sugar transporters being antiporters that require monophosphate nucleotides (such as UMP or GMP) in order to transport the activated sugar from the cytosol (121) (Fig. 3). In support of this, inhibition of GDPase in yeast causes defects in N-glycosylation (122). In addition, local cytosolic concentration of nucleotide sugar donors is controlled by enzymes that degrade nucleotide sugars, such as pyrophosphatases for UDP/GDP–sugar groups (123) and a specific hydrolase for CMP-Sia (124), which is thought to act as a way to regulate import of these nucleotide sugars by antiporters.

Supplementation of tissue slices with dolichylphosphate promotes core glycosylation generally, indicating that this important N-glycan precursor may not be saturated within the cell (125). CDG type I, which results in a global decrease in N-glycosylation levels, can be caused by a defect in the phosphomannomutase that converts mannose-6-phosphate into mannose-1-phosphate, a vital precursor for the formation of the sugar nucleotide donor GDP-Man (126, 127). However, nucleotide sugar availability cannot be a sufficient explanation for the observed N-glycan microheterogeneity. When a glycoprotein with multiple N-glycan sites passes through the secretory pathway, not all these sites are processed in the same way despite passing through the same subcellular space, and presumably through the same concentrations of nucleotide sugars. For similar reasons, theavailability of divalent cofactors is also unlikely to be responsible for the observed microheterogeneity. Thus, while precursors may impact glycosylation globally, especially in pathophysiological conditions, they are unlikely to be drivers of site-specific microheterogeneity.

### Enzyme Availability

The potential diversity of N-glycans observed is a product of the repertoire of glycan-processing enzymes that are expressed by the cell. In humans, RNases isolated from different tissues have slightly different glycan occupancies (128). Specific inhibition of glycosyltransferases contributes to changes in glycosylation seen during development. Inhibition of MGAT1 by GnT1IP promotes the production of oligomannose species during spermatogenesis (129). Studies with Lec1 cell lines, which are deficient in MGAT1, have been critical in understanding the impact of complex N-glycan formation and branching (130). By using cooler culture conditions to slow the trafficking of proteins through the secretory pathway, it is possible to increase the amount of poly-LacNAc groups on lysosomal membrane glycoproteins, presumably *via* an increase in incubation time with *trans-*Golgi enzymes (131). However, while the repertoire of glycan processing enzymes dictates the variety of structures that are possible in a cell, it is unlikely that their availability has an impact on differences in microheterogeneity between different sites on the same protein, assuming that all sites are equally accessible.

### Development

There is much interest in using glycans as biomarkers for disease, and thus, it is important to consider how N-glycan diversity is affected by age and disease state. There is a particular interest in biomarker availability in serum because of its ease of access and noninvasiveness (132). Galactosylation of human IgG fluctuates based on the age of the donor, among multiple other factors, indicating that site-specific glycosylation can change over the life of an individual (133, 134, 135). An important point to remember is that glycoproteins from serum will generally be biased toward sialylated structures, as nonsialylated structures are continuously removed from circulation by the asialoglycoprotein receptor (136, 137). Thus, the steady-state level of glycosylation in serum, while biologically relevant, is not necessarily an accurate representation of the glycoprotein population that is actually synthesized throughout the rest of an organism. Another challenge in site-specific glycopeptide analysis is a well-developed understanding of the normal range of site-specific glycosylation across healthy patients of varying ages, sexes, and ethnic groups. Developing a deeper understanding of these control groups will require close collaboration with clinicians and collection of additional data that will impact glycan processing, such as blood group typing (138).

### Disease State

Site-specific N-glycosylation can have functional consequences in physiology and disease. The abundant urine glycoprotein uromodulin has eight sites of N-glycosylation, six of which are processed to complex structures and two of which remain oligomannose. Uromodulin forms nanometer-scale filaments, and it has been found that only the oligomannose N-glycan on the filamentous arm is recognized by *Escherichia coli* adhesins, which aggregate on the filaments and are dispelled to prevent urinary tract infections (139). Patients with rheumatoid arthritis have been found to have reduced levels of IgG galactosylation, which is also correlated with disease severity (140). NMR studies of agalactosylated IgG glycans demonstrate that in the absence of Gal, the underlying glycan is liberated from glycan–protein interactions and adopts a more flexible conformation that allows the terminal GlcNAcs to be recognized by the complement-activating lectin mannose-binding protein (141). In addition, individuals with sepsis have been found to have an increase in fetuin fucosylation (142).

The impact of disease states on N-glycan microheterogeneity varies depending on the disease and must be assessed on a case-by-case basis. These changes in N-glycan microheterogeneity are intriguing in that they create opportunities for biomarker identification, particularly in cases where changes in glycosylation are important for disease progression, such as cancer (143).

### Glycan Flexibility

Glycans are flexible molecules and exist as a distribution of various conformations. N-glycans on *N*-methyl-d-aspartate receptors influence the protein structure by stabilizing an “open-clamshell” conformation that is important in facilitating transport (144). Further studies with NMR and MS confirmed this finding and also found that processing of two of the N-glycans is restricted by these glycan–protein contacts (145). Recent evidence from studies of the bacterial glycosidase EndoS suggests that the N-glycan in IgG heavy chains is able to adopt a “flipped-out” conformation that may explain its ability to mature into complex structures despite spatial restrictions (146).

Therefore, it is important to consider the glycan not as a static tree of linked sugars, but as a distribution of different conformations, only some of which may have functional importance. Due to this inherent flexibility, crystal structures often do not provide much information on the position of the glycan. Molecular dynamics studies can be a useful method of exploring the structure–function properties of glycans that are not observable *via* crystallographic methods (147), especially when used in concert with site-specific MS methods (148). However, it should be noted that most molecular dynamics studies are often limited to nanosecond to low-microsecond simulations, and thus, conformational changes that occur over longer time scales may not be observed, although continuous advances in computing are beginning to extend this range to longer time scales (149).

### Glycan Proximity Interference Interactions

In some cases, patches of N-glycosylation are so dense that neighboring glycans can themselves sterically hinder glycan processing. The most well studied of these is the case of the HIV-1 Env glycoprotein. HIV-1 Env is a densely glycosylated viral glycoprotein that is initially produced as a gp160 precursor in the ER before the molecule is processed into a trimer of gp120–gp41 heterodimers. Importantly, the glycan processing of Env is dependent on both the presence of other, proximal glycans as well as the quaternary structure of the trimer. It has been demonstrated that mutation of the N332 site to alanine increases the processing of oligomannose Env glycans (150). However, the position of the glycan is important for this effect: in contrast, mutation of sequons in the less densely glycosylated CD4 binding site had only slight effects on site-specific glycan processing (151). Additional evidence is provided by the trimer-specific formation of the trimer-associated mannose patch, which only forms when Env is processed as a trimer because of interprotomer glycan proximity interference and steric shielding. Thus, glycan proximity interference interactions can greatly influence site-specific N-glycan processing, but this may be limited to regions of dense glycosylation (such as those found on viral glycoproteins) where these glycans are in close proximity (150, 152).

Thus, while enzyme and substrate availability in a specific organism and/or cell type in a given condition define what is possible, the local three-dimensional structure near the individual sites of modification, especially on the same glycoprotein, appears to be the primary driver of observed microheterogeneity.

## Site-Specific Analysis of N-Glycans *Via* MS

### Bottom–Up Glycoproteomics—Enrichment of Glycoproteins and Glycopeptides

**Fig. 4:**
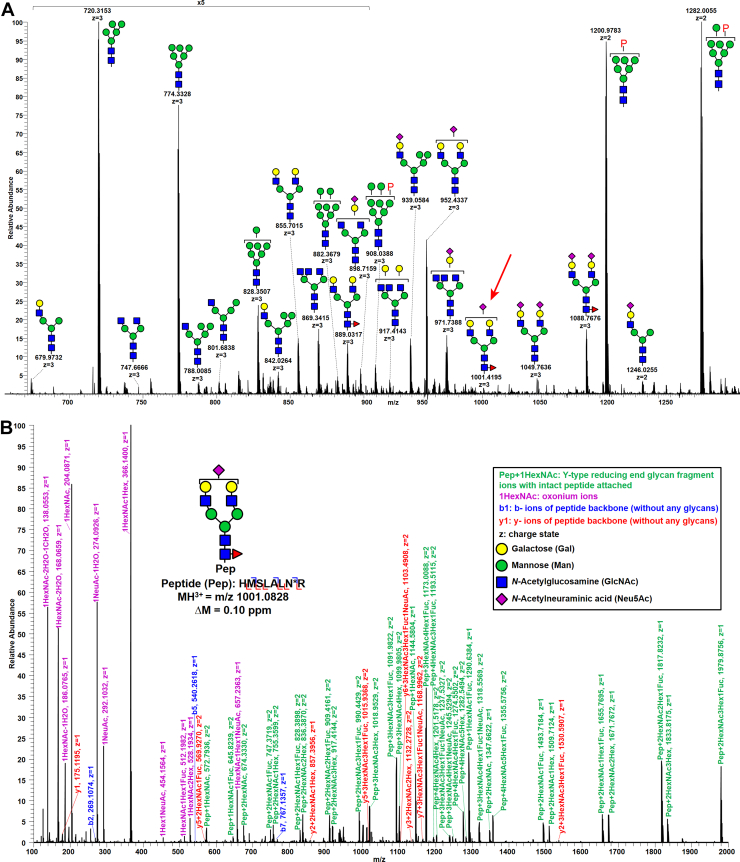
**N-glycan microheterogeneity at a single sequon**. *A*, the presented spectrum was merged from two sets of MS1 spectra averaged from different periods of retention times of a Fabrazyme peptide: (1) 48 MS1 spectra averaged from retention time 94 through 97 min; (2) 15 MS1 spectra averaged from retention time 103 through 104 min. Brackets indicate that the precise branch placement of a terminal residue cannot be confirmed, “P” indicates a phosphate group. *B*, the annotated stepped HCD spectrum of the glycopeptide species at *m/z* 1001 in *A* representing the Fabrazyme peptide modified by a single sialylated biantennary glycan with core fucosylation (Neu5Ac_1_Gal_2_GlcNAc_2_Man_3_GlcNAc_2_Fuc_1_). HCD, higher energy collisional dissociation.

Bottom–up glycoproteomic analysis by MS presents challenges in sample preparation, MS instrumentation, and data analysis because of the complexity of glycopeptides introduced by glycan heterogeneity (Fig. 4A). Traditional approaches in bottom–up glycoproteomics often involve partial or total deglycosylation of glycoproteins and glycopeptides followed by separate analyses of the protein (proteomics) (65, 105, 151, 153, 154, 155) and glycan entities (glycomics) (156, 157, 158, 159, 160, 161), which benefits from retaining linkage information, but information on site-specific glycosylation becomes lost. The analysis of intact glycopeptides allows one to investigate the site-specific variations directly. However, it also introduces significant challenges because of the underlying heterogeneity, that is, the glycoforms per definition are present in substoichiometric quantities. For this reason and others, enrichment of glycopeptides is often imperative when analyzing glycoproteins with low glycan occupancy, high site-specific complexity, or a complex mixture of glycoproteins and nonglycoproteins. Following the digestion of the glycoproteins or protein mixtures by single or multiple enzymes, intact glycopeptides can be separated and/or enriched *via* affinity chromatography, chemical properties–based interactions, click chemistry–based methods, ion mobility, and covalent chemical coupling.

The most well-established affinity chromatography approach in glycoproteomics is the use of lectins. Targeting specific glycoforms can be achieved by choosing the appropriate lectins (14, 154, 162, 163, 164, 165, 166). The most common format of lectin affinity chromatography is the use of combinations of multiple lectins to enrich for various glycoforms from the same sample (167, 168, 169, 170). Antibodies with glycosylation epitopes have also been used for glycoprotein enrichment (171, 172, 173, 174, 175, 176, 177, 178, 179, 180, 181, 182). Immobilized metal/metal oxide affinity chromatography is another commonly used approach to enrich glycopeptides. Chelating transition metal cations (Fe, Ga, Ti, Zr, etc.) and transition metal oxide (TiO_2_) not only have affinity for the deprotonated oxygens in phosphate groups but also for the carboxylic acid anions and hydroxyl groups in Sias. Applications have been reported on the enrichment of sialylated glycopeptides as well as phosphorylated glycans such as mannose-6-phosphate (183, 184, 185, 186, 187, 188, 189, 190, 191, 192, 193, 194).

Taking advantage of the hydrophilic properties of glycans, hydrophilic interaction chromatography (HILIC) has become an important tool in enriching and separating intact glycopeptides (195, 196, 197, 198, 199, 200, 201, 202, 203, 204, 205, 206, 207, 208, 209, 210, 211, 212, 213, 214, 215, 216, 217, 218, 219). Mixed-mode HILIC approaches have also become popular in recent years. Electrostatic repulsion–hydrophilic interaction chromatography, where an ion exchange stationary phase is used in combination with HILIC mobile phase, has been used to enrich for charged glycans (sialylated, sulfated, etc.) (220, 221, 222, 223, 224, 225, 226, 227, 228, 229). Porous graphitic carbon is an alternative chromatography stationary phase that enables retention of polar and hydrophilic species. It has been used in solid-phase extraction mode as well as in combination with reverse-phase liquid chromatography for glycopeptide enrichment (230, 231, 232, 233, 234, 235, 236, 237, 238, 239).

Incorporation of bio-orthogonal labels into glycans *via* click chemistry has also been used to enrich glycopeptides. This approach can be accomplished metabolically by introducing chemically functionalized monosaccharides (such as azide-sugars) into glycan biosynthesis pathways (240, 241, 242, 243, 244, 245, 246, 247, 248, 249, 250, 251, 252, 253, 254, 255, 256, 257) or chemoenzymatically, where enzymes extend the native glycans with functionalized monosaccharides (258, 259, 260, 261, 262, 263, 264, 265, 266, 267, 268).

Ion mobility chromatography separates ions based on their mobility in a carrier buffer gas and can be coupled with MS for detection. This is particularly useful in separating structural isomers of glycans and glycopeptides (269, 270, 271, 272, 273, 274, 275, 276, 277, 278, 279, 280, 281, 282, 283, 284, 285, 286, 287, 288, 289, 290). High-field asymmetric waveform ion mobility spectrometry (IMS) (291, 292, 293), also known as differential ion mobility, is a technique that is comparable to the conventional IMS and separates ions based on their motion induced by electric fields at atmospheric pressure and room temperature. Often coupled with electrospray ionization, field asymmetric waveform IMS is typically used as an ion filter placed between the ion source and the mass analyzers for an additional level of separation of isomeric glycopeptides (135, 272, 280, 284, 285, 286, 287, 288, 289), although it does not directly measure the collisional cross-section values for individual ions (280, 294, 295, 296). Trapped IMS in tandem with stepped collisional energies (SCEs) has been shown to be particularly effective in enhancing glycopeptide and glycoprotein identification (297, 298). Drift tube ion mobility MS could potentially be used to separate and analyze structural isomers, as current glycopeptide analysis workflows are often limited to describing compositions of glycans rather than distinct structures because of identical *m/z* and similar chromatographic profiles. While promising, drift tube ion mobility MS still has much room for development before becoming broadly useful, as confident analysis will require difficult-to-synthesize standards, and computational collisional cross-section predictions for even free glycans are still a work in progress (299, 300, 301, 302, 303).

Other approaches, such as covalent chemical coupling *via* hydrazide (304, 305, 306, 307, 308, 309, 310, 311, 312, 313, 314, 315, 316) and boronic acid (317, 318, 319, 320, 321, 322, 323, 324, 325) are also frequently used to enrich and separate glycopeptides. However, those methods rely on deglycosylation to release the enriched and separated glycopeptides and therefore are not often used for intact glycopeptide analysis. We would like to direct our readers to dedicated reviews for more detailed and in-depth discussion on the enrichment techniques of glycopeptides (326, 327, 328).

### Bottom–Up Glycoproteomics—Fragmentation and Instrumentation

Advances of MS have allowed analysis of intact glycopeptides, where the sequences of peptide backbones and glycan moieties can be obtained simultaneously. Undergoing collision-based fragmentation, such as collision-induced dissociation (CID), glycopeptides can be fragmented to produce both peptide backbones (b- and y-type ions of peptide bonds (329)) and glycan moieties (B- and Y-type ions of glycosidic bonds (330)) (331, 332, 333, 334). The glycan B-type fragments, also referred to as oxonium ions, generated by CID are often used as the diagnostic ions of glycopeptides, such as the ions of a single *N*-acetylhexosamine (HexNAc) at *m/z* 204 and a disaccharide of Hexose1HexNAc1 at *m/z* 366. Depending on how the kinetic energy is deposited into a peptide, resonant-excitation CID and beam-type CID tend to generate different patterns of product ions even though both fragmentation uses low collisional energy (CE; typically 10–100 eV per charge). Resonance CID of glycopeptides often produces abundant glycan fragments but lacks peptide fragments because of the vibrational energy breaking the most labile bonds first (glycosidic bonds in the case of glycopeptides), leaving the remaining of the peptide backbone underenergized for dissociation. To resolve this issue and further characterize the peptide backbone of glycopeptides, multistage CID has been utilized to produce necessary backbone fragments for peptide sequencing (332, 335, 336, 337, 338). Beam-type CID, originally developed in triple quadrupole and quadrupole time-of-flight instruments (339), occurs at a slightly higher energy level than resonance CID, and its fragment ions can retain higher vibrational energy, leading to further dissociation. Therefore, beam-type CID of glycopeptides can produce both glycan and peptide fragments that are useful for sequencing (340). Later, beam-type CID was implemented in hybrid ion trap–Orbitrap mass spectrometers, often termed high-energy C-trap dissociation or higher-energy collisional dissociation (HCD) (341). However, optimizing for a single value of CE of CID or HCD that is universally suited for fragmenting a wide range of glycopeptides differing in glycan moieties, peptide sequences, and charge states, to yield the highly informative Y-type reducing-end glycan fragments as well as b- and y-type peptide backbone fragments, remained challenging. Hinneburg *et al*. (342)used synthetic N-glycopeptides to systematically optimize CID energy parameters on quadrupole time-of-flight instruments. Cao *et al*. (236)evaluated individual HCD CEs for fragmenting intact glycopeptides and found different optimal CE values for glycan moieties and peptide backbones. Later on, the option of SCE became available in Orbitrap instruments, where a central value and a variation value are defined by users to obtain three CE values. Using HCD with SCE, or SCE HCD, the same precursor ions are injected three times and fragmented with the three CEs correspondingly, the product ions from three fragmentation are combined and temporarily stored in the C-trap and then sent to a user-selected analyzer for detection resulting a multiplexed spectrum where B-type glycan oxonium ions and b-/y-type peptide backbone ions produced at high CEs coexist with Y-type reducing-end glycan ions produced at low CEs (Fig. 4B). The strategy of stepped HCD has been widely adopted and applied in intact glycopeptide analysis (90, 148, 343, 344). A more versatile feature of CE selection mode has been developed recently to facilitate the fragmentation of small molecules, the assisted CE, where a single optimal energy would be selected in real time from a list of energies input by users (345). Alternatively, high-energy CID with kinetic energy at 1 to 10 keV has also been used to produce glycan and peptide fragments simultaneously (346, 347, 348, 349).

While CID inherently generates peptide backbone fragments with the loss of glycan moieties, thelocalization of the glycosylation sites cannot be readily achieved in most cases, especially when multiple sites of glycosylation coexist on a single peptide. Radical-induced fragmentation approaches, such as electron transfer dissociation (ETD) (350) and electron capture dissociation (ECD) (351), produce peptide backbone fragments with any labile modification attached, therefore enabling unambiguous site localization (352, 353, 354). Several hybrid instrument methods have been developed combining CID and ETD for a comprehensive characterization of intact glycopeptides (202, 355, 356). To further complement ion activation techniques such as CID and ETD in tackling singly and doubly charged analytes that cannot produce detectable fragment ions under ETD or ECD, high-energy electron-activated dissociation techniques, such as electronic excitation dissociation (EED), electron impact excitation of ions from organics, and electron-induced dissociation, have also been used for analyzing glycoconjugates (357, 358, 359). Zhao *et al*. (360)combined HCD and ETD for the analysis of O-linked glycosylation, which led to the implementation of HCD product ion–triggered ETD in the hybrid ion trap–Orbitrap mass spectrometers from Thermo Scientific. Since then, the technique of HCD production–triggered ETD has been used to characterize intact glycopeptides, achieving glycan moiety sequencing and site localization in a single LC–MS/MS analysis (162, 361, 362, 363, 364, 365, 366, 367).

The Omnitrap platform has been introduced recently as a multidimensional multistage tandem ion processing system that is designed as a segmented linear ion trap where the ion trapping is performed by rectangular waveforms, and ion transferring between trapping regions is enabled by fast switching direct current potentials. This novel platform provides access to a variety of ion fragmentation techniques, including slow heating and beam-type CID, electron-based dissociation (ETD, ECD, electron-induced dissociation, and EDD), photodissociation (UV photodissociation and infrared multiphoton dissociation), and additional activation methods enabled by external injection of activated neutrals or pulsed ion beams (368, 369, 370). Wei *et al*. (371)reported detailed glycan structural characterization by EED on a hybrid Orbitrap–Omnitrap instrument and established *de novo* glycan sequencing by a novel EED MS^2^-guided MS^3^ strategy. Li *et al*. (372)conducted site-specific intact glycopeptide analysis on a hybrid Orbitrap–Omnitrap platform and demonstrated the exceptional performance of high-energy electron-activated dissociation, EED particularly, for unambiguous site localization, peptide backbone sequencing, as well as glycan topology and linkage elucidation. Linkage elucidation historically has required glycomic analyses on released glycans, thus, the promise of potentially being able to determine glycan structure beyond topology *via* glycoproteomics is extremely exciting and would represent a major step forward in the field.

### Bottom–Up Glycoproteomics—Data Analysis

Advances in dedicated software solutions and bioinformatics tools for high-throughput glycopeptide identification have aided the automation of glycoproteomic analysis in recent years (199, 226, 373, 374, 375, 376, 377, 378, 379, 380, 381). However, challenges remain, particularly for the quality control of database search output, where a false discovery rate (FDR) evaluation is needed for both glycan parts and peptide parts of the glycopeptide matches. For peptide identification, the target-decoy strategy is usually employed to estimate the peptide and protein FDR, and a sequence-based decoy database generated from the target database is routinely used. Unlike homogenous peptides, glycans are often heterogeneous branched trees because of their non–template-driven synthesis, which creates difficulties in constructing a corresponding tree-based target-decoy database that is necessary for FDR estimation. The use of an FDR for the glycan assignment increases the specificity and sensitivity in identifying complex intact glycopeptides and allows for FDR control of both the peptides and glycans. However, most available software currently only calculates FDR on peptides but not on glycans (199, 226, 373, 375, 378). There are only a few exceptions where the glycan FDR is considered.

pGlyco addressed the glycan FDR issue by introducing a spectrum-based target-decoy method to estimate glycan FDR of glycopeptides (379). In this method, the theoretical target glycopeptide spectrum was first generated after the masses of the Y-type glycan reducing-end ions (with intact peptide backbone attached) were deduced based on the putative peptide backbone mass, then a random mass ranging from 1 to 30 Da was added to the mass of each deduced Y-type ion to generate a theoretical decoy spectrum, and the glycan FDR for the glycopeptides was calculated based on matches against both databases. A finite mixture model was also employed to adjust the bias from assuming “the number of incorrect identifications from target or decoy sequences are equally likely” (382) in a target-decoy approach (379, 383).

In newer versions of the software, MSFragger (376) also addressed the glycan FDR issue for N-linked glycopeptides by introducing an associated glycan FDR estimation method, which includes both Y- and B-type glycan fragments (384). Their new glycan FDR estimation approach first matches the peptide sequence, then matches the mass difference between the peptide sequence mass and the observed precursor mass to candidate glycans to determine the composition. After that, pairwise comparison of all candidates determines the best match to the spectrum based on unique fragment ions for each candidate and mass and isotope errors. Finally, a composite glycan score is generated from a variety of spectral evidence, including Y-ions, B-ions (oxonium ions), and the observed mass and precursor isotope errors, and FDR is computed using the score distribution of target and decoy glycans (376, 384).

A recent multilaboratory evaluation of existing software and informatics solutions for large-scale glycopeptide analysis revealed comparable performance of freeware and commercial products, with similar limitations, especially for matching glycans with similar or identical masses (*N*-acetylneuraminic acid, *N*-glycolylneuraminic acid, multi-Fuc, methionine oxidation, and cysteine carbamidomethylation) (385). Isomeric or isobaric glycopeptides with or without oxidation and alkylation are often misassigned by the evaluated search engines, demonstrating the need for improvement in matching isobaric and near-isobaric glycopeptides. The search outputs in terms of specificity (accuracy) and sensitivity (coverage) were also variable with different search engines, indicating that orthogonal searches and a pool of results could be useful for a comprehensive glycoproteomics analysis. The search parameter settings also contributed to the discrepancy associated with search results, especially the post-processing tools used to filter search results (385). In the case where N-linked and O-linked glycosylation coexist on the same peptide sequence, the inefficiency of current software solutions becomes even more pronounced. For these reasons, automated analysis of glycopeptide structures remains challenging. In the meantime, in order to increase the accuracy of glycopeptide and glycoprotein analysis especially for reporting, we recommend (1) using multiple search engines to analyze the same dataset, (2) performing separate searches targeting N- and O-linked glycosylation, especially for glycopeptide/glycoproteins that are known or suspected to be both N- and O-glycosylated, and (3) validating search engine outputs by manual examination of the raw spectra. Given the complexity of glycopeptide data and the incapability of current software solutions, manual validation, while time intensive and quantitatively not satisfying, is often still necessary to ensure the accuracy of glycopeptide assignment.

### Utilization of Mixed Workflows and Glycosidases

While glycoproteomics can provide site-specific glycan information and general glycan topology, it can be difficult to differentiate between the compositions of specific glycan antennae since most of the structural and linkage information cannot be obtained from single-stage fragmentation of underivatized glycopeptides. MS-driven glycoproteomics can also be facilitated by glycomics, often known as glycomics-assisted or glycomics-informed glycoproteomics (148). In a glycomics-informed glycoproteomics study, the profile of glycans is released by enzymatic treatment or chemical reaction and analyzed for their linkage and structural details that is often unattainable or ambiguous from the analysis of intact and underivatized glycopeptides. This is especially useful for the elucidation of terminal and internal or core structures, such as differentiating core and terminal fucosylation, which is often ambiguous in intact glycopeptide analysis since both features produce the same B-type glycan oxonium ions and the specific Y-type ions that can differentiate between them are often elusive. Not only does glycomics provide additional structural information and an orthogonal validation to the glycopeptide analysis but also it helps expand the application of glycoproteomics to complex biological samples, such as protein extracts from cells, tissues, and bodily fluids (386). Alternatively, similar to its use in glycomics, chemical derivatization can often improve the ionization of glycopeptides as well as produce more informative fragment ions for glycan structural and linkage determination. Recent development on analyzing intact glycopeptides *via* esterification has been reported (387, 388, 389). Novel derivatization method such as methylamidation was applied to analyze the structures of N-linked glycopeptides (390), and a hybrid workflow of differential chemical modification has also been reported (195).

Linkage-specific glycosidases can also be a useful tool in elucidating glycan structures with greater specificity. An example of this can be seen with the determination of Sia linkages in complex N-glycans. Sias on N-glycans are usually found in either an ⍺-2,6 or an ⍺-2,3 linkage. Since fragmentation of underivatized glycopeptides cannot provide enough information to distinguish between these two different linkages, linkage-specific sialidases can be used to treat the glycopeptides, and their respective enzymatic products can be used to determine the identity of specific sialylated structures (391). While most sialidases are either ⍺-2,3 specific or of a broad ⍺-2,3/⍺-2,6/⍺-2,8 specificity, the recent development of an ⍺-2,6-specific sialidase may be able to further power these kinds of analyses (392).

A method for determining the relative ratios of occupancy and N-glycan classes while retaining site specificity is described by Cao *et al*. (151, 393). This approach utilizes the differing specificities of endoglycosidases and comprises the sequential use of Endo H that removes oligomannose and hybrid N-glycans, and peptide N-glycosidase F in the presence of ^18^O-water that removes any type of mammalian N-linked glycans other than the product of Endo H. The end result is that sequon-containing peptides will exist in some or all three forms that denote their occupancy: (1) Asn-GlcNAc (+204 Da difference, oligomannose/hybrid); (2) ^18^O-Asp (+3 Da difference, complex); and (3) unmodified (+0 Da difference, unoccupied). Even though this approach can only provide information on the general glycan types (oligomannose/hybrid, or complex) and not the specific glycoforms occupying each site, a major benefit of this approach is that for glycopeptides that have the same peptide backbones, it reduces the heterogeneity of the glycopeptide population into three distinct subpopulations, which coalesces the signal intensity, thus reducing the required sensitivity of MS instrumentation. In addition, it reduces the differences in ionization between differing glycopeptide species and therefore, theoretically, streamlines quantitation.

### Other Approaches

Other than inferring from bottom–up glycopeptide fragments, analysis of intact glycoproteins by top-down MS can complement the bottom–up approach. Though analysis usually loses details on site-specific glycosylation and is often more demanding in instrumentation and data analysis, the top–down approach provides information on the upper levels of protein organization and protein complexes that normally cannot be observed by the bottom–up approach, such as glycoprotein structural heterogeneity, conformation, and dynamics. Several groups have reported their findings in analyzing intact glycoproteins by top–down MS in denaturing or native conditions or a hybrid approach of both (142, 394, 395, 396, 397, 398, 399, 400, 401, 402, 403, 404, 405, 406, 407, 408). Wu and Robinson (409)have reviewed recent progress in employing native MS for elucidating structural heterogeneity and biomolecule function of intact N- and O-linked glycoproteins. These top–down approaches will be particularly useful in determining the effects of “asymmetrical glycosylation” of IgGs, which have been shown to have some impacts on immune function (410).

## Conclusion

Microheterogeneity is an inherent characteristic of N-glycoproteins, and in order to understand these biomolecules in their entirety, this diversity needs to be considered. N-glycans have been shown to impact glycoprotein function through both the actions of specific glycoforms and through the general properties of N-glycans, including modulating the actions of many biologics. Site-specific glycosylation can be altered in disease states, and much work still needs to be done to develop useful site-specific glycan biomarkers for early detection of diseases such as cancer (138, 411).

Bottom–up glycoproteomics is a direct way to characterize site-specific N-glycan diversity. This is a method that is still rapidly developing as both analytical and computational technologies improve to meet the demanding accuracy, specificity, and sensitivity needed for glycoprotein analyses. However, this method often needs to be complemented by other methods, such as glycomics and glycosidase treatment, to gain a more comprehensive understanding of linkage information and relative glycan quantities. Methods outside MS that interrogate the structure of glycoproteins, such as crystallography, cryo-EM, NMR, and molecular dynamics, can all be utilized to more fully understand the role of the glycan in the context of the glycoprotein.

Advances in understanding what causes a glycoform to be enriched at a specific site are critical for further advances in “glycoengineering” biologics to generate more defined and consistent N-glycans. In addition, improved front-end, instrument, and back-end software workflows are needed to make glycoprotein analyses more routine and to begin to address quantification. Artificial intelligence/machine learning tools offer exciting opportunities in driving the ability to predict microheterogeneity as well as improving assignment protocols from glycoproteomic data. As our technology and understanding of N-glycans grow, look to this field for exciting new opportunities in our understanding of various pathophysiological conditions and in the development and improvement of biologic-based therapeutics.

## Conflict of interest

The authors declare no competing interests.
